# The effects of continuous oromotor activity on speech motor learning: speech biomechanics and neurophysiologic correlates

**DOI:** 10.1007/s00221-021-06206-5

**Published:** 2021-09-15

**Authors:** Kaila L. Stipancic, Yi-Ling Kuo, Amanda Miller, Hayden M. Ventresca, Dagmar Sternad, Teresa J. Kimberley, Jordan R. Green

**Affiliations:** 1 Department of Communicative Disorders and Sciences, University at Buffalo, Buffalo, NY, USA; 2 Department of Physical Therapy, Upstate Medical University, Syracuse, NY, USA; 3 Department of Communication Sciences and Disorders, MGH Institute of Health Professions, Boston, MA, USA; 4 Department of Rehabilitation Sciences, MGH Institute of Health Professions, Building 79/96, 2nd Floor 13th Street, Boston, MA 02129, USA; 5 Department of Biology, Northeastern University, Boston, MA, USA

**Keywords:** Transcranial magnetic stimulation, Speech, Motor learning, Chewing, Cortical silent period, Bulbar

## Abstract

Sustained limb motor activity has been used as a therapeutic tool for improving rehabilitation outcomes and is thought to be mediated by neuroplastic changes associated with activity-induced cortical excitability. Although prior research has reported enhancing effects of continuous chewing and swallowing activity on learning, the potential beneficial effects of sustained oromotor activity on speech improvements is not well-documented. This exploratory study was designed to examine the effects of continuous oromotor activity on subsequent speech learning. Twenty neurologically healthy young adults engaged in periods of continuous chewing and speech after which they completed a novel speech motor learning task. The motor learning task was designed to elicit improvements in accuracy and efficiency of speech performance across repetitions of eight-syllable nonwords. In addition, transcranial magnetic stimulation was used to measure the cortical silent period (cSP) of the lip motor cortex before and after the periods of continuous oromotor behaviors. All repetitions of the nonword task were recorded acoustically and kinematically using a three-dimensional motion capture system. Productions were analyzed for accuracy and duration, as well as lip movement distance and speed. A control condition estimated baseline improvement rates in speech performance. Results revealed improved speech performance following 10 min of chewing. In contrast, speech performance following 10 min of continuous speech was degraded. There was no change in the cSP as a result of either oromotor activity. The clinical implications of these findings are discussed in the context of speech rehabilitation and neuromodulation.

## Introduction

Impairments in the ability to produce understandable and natural speech are caused by a variety of neurologic abnormalities (Duffy 2013) and are well-known to negatively impact quality of life (McAuliffe et al. 2017; Piacentini et al. 2014; Walshe and Miller 2011). Although therapies for improving the intelligibility and quality of speech are available (see Duffy 2013; Finch et al. 2020; Palmer and Enderby 2007; Yorkston 2010 for overviews of available therapies), current interventions have limited effectiveness and often, do not result in complete recovery of intelligible and natural-sounding speech (Herd et al. 2012; Mitchell et al. 2017). In general, the field of speech-language pathology could benefit from more effective, efficient, and long-lasting therapies for improving speech in a variety of patient populations. Therefore, the search for expeditious and sustainable speech treatments is a high research priority.

For decades, research on neuroplasticity has focused on identifying ways to induce an optimal neurochemical environment for enhancing learning and rehabilitation of cognitive, language, and motor disorders. Most of this research has focused on pharmacologic agents (Tran et al. 2016) or extrinsic neuromodulation techniques such as repetitive transcranial magnetic stimulation (rTMS) or transcranial direct current stimulation (tDCS). Extrinsic neuromodulation has been found to improve rehabilitation of limb motor function in a variety of patient populations (Benabid et al. 2009; Borich et al. 2009; Brien et al. 2018; Kimberley et al. 2013, 2018; Wu et al. 2008). Previous literature has also demonstrated the potential for central and peripheral stimulation to have beneficial effects on the bulbar (i.e., head and neck) musculature via neuroplastic changes in primary motor cortex (Boliek and Fox 2014; Doeltgen et al. 2010; Pisegna et al. 2016; Rofes et al. 2013). Arguably, the strongest evidence comes from studies on neuromodulation techniques including peripheral electrical stimulation (Byeon and Koh 2016; Chen et al. 2016; Doeltgen and Huckabee 2012; Hamdy 2010; Robbins et al. 2008; Sassegbon et al. 2020), tDCS (Doeltgen et al. 2015; Pisegna et al. 2016; Sassegbon et al. 2020), and rTMS (Pisegna et al. 2016; Sassegbon et al. 2020), that improve swallowing outcomes in individuals with neurogenic dysphagia. Additionally, a small number of studies have shown promising benefits of tDCS on speech production of healthy individuals (Buchwald et al. 2019) and in individuals with apraxia of speech (Wang et al. 2019), though more research in these areas is greatly needed.

In limb motor control, an increasing number of studies have evaluated activity-based methods for inducing neuroplastic changes to enhance limb motor recovery through dynamic or sustained motor activity. The therapeutic benefits of continuous motor activity on limb motor function has been demonstrated in both healthy individuals and in individuals with neurologic diseases (Fisher et al. 2008; Hirsch et al. 2016; Petzinger et al. 2013). For example, Fisher et al. (2008) found that a treadmill training exercise protocol both modified cortical excitability and improved parameters of gait (i.e., speed, stride length, weight distribution, etc.) in participants with Parkinson’s disease (PD). The premise of the approach is that specific motor activities induce cortical excitability that is subsequently harnessed to enhance learning (Petzinger et al. 2013). The enhancing effects of neuromodulatory agents, including activity-induced excitability, have been referred to by a few authors as “priming the brain” to optimize response to therapy (Cassidy et al. 2015; Dietsch et al. 2019; Ward 2016). Presumably, the candidate priming activity has short-term lasting effects (e.g., 10–20 min) on neuroplasticity, such that it can be performed prior to working on the treatment target. Thus, the mechanism for enhancing learning for priming activities are distinct from those elicited during task-specific approaches to motor practice, which require parity between the physiological demands of the practiced task and the treatment target (Kleim and Jones 2008; Krakauer 2006). For example, when employing speech rehabilitation techniques that adhere to the principles of task-specificity, one would practice speech or speech-like behaviors and not a behavior like chewing, which has very distinct physiologic demands from those of speech (Moore et al. 1988).

Activity-induced excitability is an appealing therapeutic modality because it is simple and inexpensive, and conceivably provides more relevant targets because it engages patients’ own behavior to activate applicable brain regions. In the current study, we tested the novel hypothesis that continuous oromotor activities (i.e., 10 min of chewing and speech), would impact subsequent speech learning via changes to cortical excitability, and that the learning effect would differ depending on the type of oromotor activity. Chewing and speech were of interest because they are activities that most individuals engage in in everyday life, and thus, would provide a baseline level of activity-induced neuroplasticity that the corticobulbar system is capable of.

To our knowledge, the efficacy of continuous oromotor activity for speech rehabilitation has not been previously considered, although prior research from several fields (e.g., dentistry, neuropsychology, etc.) have reported the enhancing effects of swallowing and chewing on learning of swallowing-specific and general cognitive tasks. Swallowing and chewing engage widespread cortical excitability and induce neuroplastic changes (Avivi-Arber et al. 2011) via increased activity in a variety of brain areas that support learning and memory, as well as areas that are important for motor control (Lin 2018; Quintero et al. 2013; Tramonti Fantozzi et al. 2017). Several studies have shown beneficial effects of swallowing on subsequent swallowing behaviors both at behavioral (i.e., improvement of swallowing function) and neural levels (i.e., enhancement of cortical excitability) in patients with dysphagia secondary to neurological damage and/or disease (Al-Toubi et al. 2011; Macrae and Humbert 2013; Macrae et al. 2014).

The beneficial effects of chewing on behavior are well-established in the animal literature (Scherder et al. 2008; Sessle et al. 2007). Chewing activity has been shown to enhance multiple processes that are critical for learning, including vigilance, attention, cognition, executive function, working memory, positive mood, recall, cognitive processing speed, and learning (Tramonti Fantozzi et al. 2017; Tucha and Koerts 2012). Conversely, reduced performance on behavioral tasks, such as mazes and avoidance tasks, has been associated with decreased masticatory activity, tooth loss, and chewing softer rather than harder foods in animals (Lin 2018). These studies on non-human animals provided a rationale for examining the effect of continuous chewing on human motor cortex and if these changes are modulated by intraoral modifications or different food textures.

The neuroplastic effects of sustained motor activity have been demonstrated primarily in studies of cortical excitability using transcranial magnetic stimulation (TMS) (Fraser et al. 2003). For example, one study reported an increase in cortical excitability in response to the learning of novel lip and jaw movement patterns (Avivi-Arber et al. 2011). Primarily, studies have examined the changes in motor evoked potentials (MEPs) as a result of a modulating activating; however, changes in the cortical silent period (cSP; which will be discussed in further detail in the methods section) has also been found to be related to improved motor learning (Ljubisavljevic 2006; Mak and Hallett 2013; Neva et al. 2017). The underlying premise of this paradigm is that motor behaviors can induce cortical excitability, which, in turn, facilitates learning or improvement of performance (Komoda et al. 2015; Zhang et al. 2016). This pathway may be one mechanism by which motor activity can reinforce learning. Although evidence for increases in oromotor cortical excitability has been established by a number of previous studies (Iida et al. 2019; Komoda et al. 2015; Kothari et al. 2013; Svensson et al. 2006), it is unclear how these neurophysiologic changes facilitate improved *functional outcomes*, such as speaking or eating, that are important for everyday life (Kothari et al. 2013; Zhang et al. 2016). Authors in the dysphagia literature have highlighted the need for functional outcomes to be included in these types of studies (Macrae and Humbert 2013). In addition, although there is literature to suggest that chewing induces cortical excitability, to our knowledge there is not a parallel literature exploring potential neuroplastic changes induced by continuous speech.

### The current study and research questions

The current exploratory study was designed to examine the short-term effects of activity-induced cortical excitability on speech performance. Specifically, we were interested in how continuous periods of chewing and speech would affect the ability of neurologically healthy young adults to perform a novel speech motor learning task. The speech motor learning task designed for this study is described in more detail in the methods section. The rationale for this task is that it engages the multiple processes required for non-word pronunciation training such as attention, memory, planning, execution, and feedback control (MacPherson 2019). Other studies have used similar ‘listen-and-repeat’ type speech tasks (Reiterer et al. 2013; Restle et al. 2012) with the assertion that this type of task would simulate the type of relearning that an individual engages in following neurologic injury or disease that causes speech motor impairments (c.f. Case and Grigos 2016; Sadagopan and Smith 2013; Whitfield and Goberman 2017b). In the current study, we defined learning as the short-term performance changes that occur over the course of many repetitions of a novel speech task.

A variety of speech outcome measures that were derived from various signals (i.e., perceptual, acoustic, and kinematic) were examined to comprehensively evaluate changes in speech performance across tasks and trials. For example, we used kinematic measures of lip speed and distance (1) to provide the physiologic context for the observed changes in speech behavior which allows for a deeper understanding of the effects, but also (2) because they are likely to offer increased sensitivity for detecting effects than more subjective measures of speech performance such as perceived accuracy. Improvements in the perceptual, acoustic, and kinematic measures across the duration of the speech learning task would be indicative of enhanced performance. The results of this work could have implications on the design of speech therapy protocols, provide baseline information on the neuroplastic capability of the healthy nervous system, and heighten our understanding of the interaction between speech and non-speech oromotor behaviors. All of these applications have been previously identified as areas of need in our field (Gonzalez Rothi et al. 2008; Ludlow et al. 2008; Martin 2009; Robbins et al. 2008; Sessle et al. 2007). This work is innovative for its use of a variety of sophisticated methodological and measurement techniques, its creation of a novel speech motor learning task, and its theoretical basis which has been understudied in the field of speech science to date. To this end, the following research questions were of interest:

1. What is the effect of continuous chewing and speech activity on the rate and extent of articulatory improvement over multiple repetitions of unfamiliar nonwords?
2. Is the rate and extent of speech performance improvement related to changes in cortical excitability?

## Methods

### Participants

Twenty young adults between the ages of 20 and 30 (mean = 24.65 ± 3.5 years; 14 females) participated in this study. This number of participants was chosen to be comparable to the number of subjects used in many other exploratory studies involving TMS measurements of cortical excitability (Boudreau et al. 2013; Chen et al. 2020; Fraser et al. 2003; Kimberley et al. 2009; Paradiso et al. 2005; Samargia et al. 2014, 2016; Sowman et al. 2014; Zhang et al. 2016). Participants all reported a negative history of speech, language, and neurological disorders and reported adequate vision to read stimuli. Participants passed a hearing screening at 500, 1000, 2000, 4000, and 8000 Hz (Hz) at 30 decibels (dB) bilaterally. All participants spoke English as their primary language and only one was bilingual (spoke Gujarati); the other 19 participants were monolingual English speakers. All participants were right-hand dominant by self-report and as scored by the Edinburgh Handedness-Inventory (Oldfield 1971). Working memory was assessed with the forward and backward digit span tasks from the Wechsler Adult Intelligence Scale (WAIS; Wechsler 2010) and all participants scored within the normal range of working memory (i.e., between six and nine on the forward digit span task (mean = 7.8 ± 0.95); between four and eight on the backward digit span task (mean = 6.1 ± 1.02)). Participants also completed a safety screening for use of TMS (Rossi et al. 2009) and all denied any history of seizure, metal in head/neck, or implanted devices (i.e., pace maker, deep brain stimulation, etc.). All participants gave written informed consent prior to initiation of study procedures. This study was approved by the Institutional Review Board (IRB Protocol Number: 2019P000326) through Mass General Brigham (MGB) in Boston, MA.

### Procedure

**Fig. 1:**
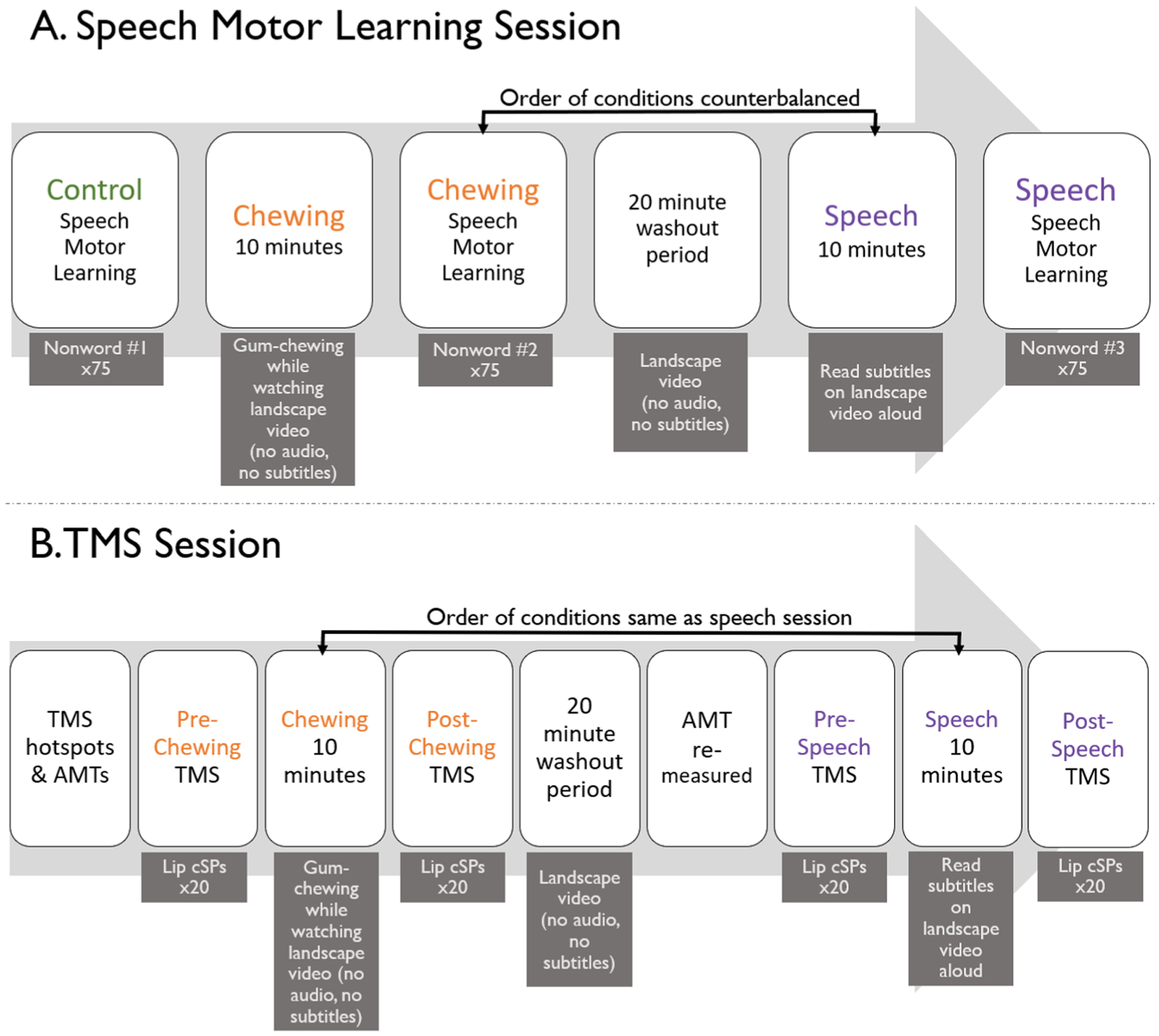
Flowchart of the study. **A** Order of experimental procedures for the speech motor learning session. **B** Order of experimental procedures for the transcranial magnetic stimulation (TMS) session. *AMT* active motor threshold

Participants completed two study sessions: one session involved participation in a speech motor learning task and the other session involved the TMS procedures described below. A flowchart of all of the experimental procedures is presented in Fig. 1.

#### Motor learning task

##### Stimuli and instructions

A novel speech motor learning task was designed for this study. The task involved participants hearing an auditory model of nonwords consisting of eight nonsense syllables (e.g., /tɑ ðu mæ zi tʃæ gi fɑ vum/). There were three eight-syllable nonwords in total. Each nonword contained eight consonant–vowel syllables (except the last syllable which was consonant–vowel-consonant in structure). Although they were not words in English, they were phonotactically possible in English. We chose eight syllables to create a challenging task that maximizes the visible learning curve and preempts any ceiling effects seen in other paradigms with healthy participants (Sasisekaran et al. 2010; Steinberg Lowe and Buchwald 2017; Whitfield and Goberman 2017a). Each syllable contained one of the four corner vowels. The nonwords began with a stop consonant and ended in a nasal consonant to aid in the parsing of the acoustic and kinematic signals. Additionally, we alternated early-developed consonants and later-developed consonants between syllables. Nonwords were controlled for phonotactic probability using an online calculator for biphone probability statistics (Vitevitch and Luce 2004). The auditory model of the nonwords was recorded by a native female speaker of standard American English. To the best of the speaker’s ability, the syllables contained an equal amount of stress and each recorded nonword was ~ 2 s in length.

Participants were given the instructions that they would hear a set of eight syllables that did not make sense. Participants were instructed to repeat the heard syllables to the best of their ability. Participants then heard the same nonword again, and they were told to repeat it again. Nonwords were presented auditorily 75 times with instructions to repeat the nonword after each auditory presentation. Participants were not able to ask for a repetition of the auditory model; therefore, if they failed to respond in ~ 5 s following a model, the experimenter moved onto the next model. The experimenter manually advanced to the next auditory model as soon as the participant finished the previous production. Participants were instructed to first prioritize the accuracy of their productions, and secondly, to prioritize speed, so that by the end of the 75 repetitions, they would be speaking as quickly as possible, possibly even faster than the auditory model they heard. They were given a short ~ 1 min break after 40 repetitions. During this break, the experimenter provided a live model of the nonword by standing in front of the participant and saying the nonword aloud, without providing feedback about the correctness of the participants’ previous productions. During pilot testing, some participants struggled to correctly produce the nonwords and for some, seeing/hearing a live model was helpful for correcting their productions. Participants then completed the last 35 repetitions in the same manner as the first 40 repetitions.

##### Procedure

A flowchart of the experimental procedures for the speech session is presented in Fig. 1A. One nonword was used for the control condition, a second nonword for the chewing condition, and a third for the speech condition. The order of nonword presentation was counterbalanced across participants. The control condition was always completed first to avoid carry-over effects from the other conditions. Participants completed the motor learning task as described above at baseline, prior to completion of any other tasks. Then, participants were instructed to chew a piece of sugar-free gum for 10 min. Previous literature has found 10 min of chewing to result in cognitive-task-performance benefits (see Onyper et al. 2011; Sakamoto et al. 2009; Tramonti Fantozzi et al. 2017). While chewing, participants were shown a nature/landscape video that did not have accompanying audio, nor did it contain animals or people. Following 10 min of chewing, participants completed the speech motor learning task again with a second nonword. A 20-min wash-out period was then given during which participants watched a different nature video, again without audio or animals/people. Because previous research has suggested that the beneficial effects of chewing on cognitive tasks persists for approximately 15–20 min after chewing has ceased (Onyper et al. 2011; Tramonti Fantozzi et al. 2017), a wash-out period of 20 min was used to eliminate the effects of one condition on the following condition. After the wash-out period, participants were instructed that they were going to speak aloud for 10 min. They were shown the same video as during the chewing condition, but this video included subtitles that participants were instructed to read aloud. The subtitles were sped to 1.25 times their original speed to provide a more challenging reading task. We used a reading task rather than conversational speech to reduce the potential cognitive load that may result from generating spontaneous speech (Wang et al. 2010), as well as to control the linguistic content and flow of speech. Following this 10-min video, participants completed the motor learning task one final time with the third nonword. The chewing and speech conditions were counterbalanced across subjects.

**Fig. 2:**
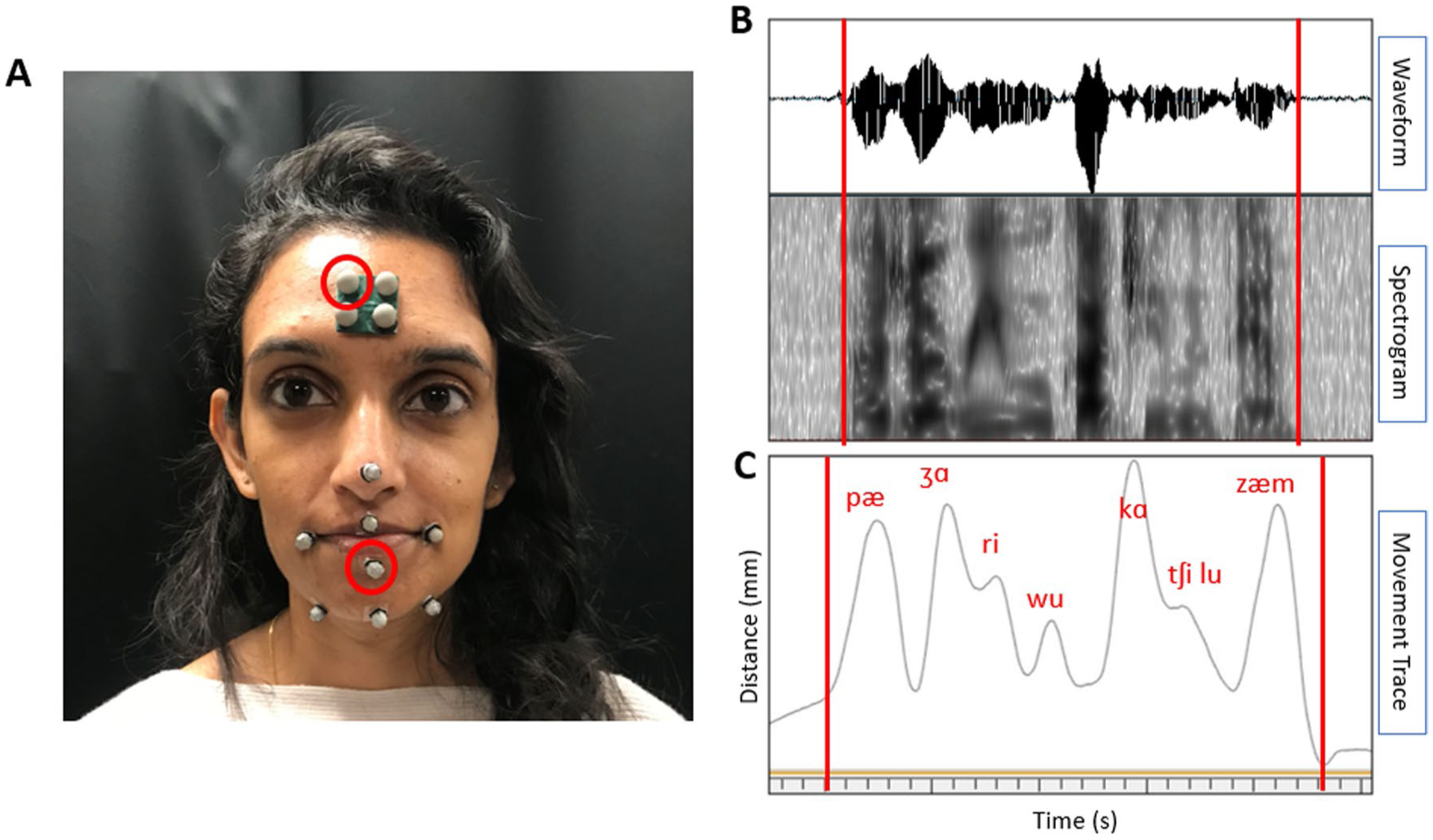
Speech motor learning data collection set-up and analysis. **A** Placement of the reflective markers for optical motion capture. The markers circled in red were of interest for the current analyses (center lower lip and right top head). **B** The waveform and spectrogram from the audio of one repetition of the speech motor learning task. The beginning and end of the repetition are marked with red lines. **C** The movement trace of the lower lip from one repetition of the speech motor learning task. The beginning and end of the repetition are marked with red lines

While completing the motor learning task, participants wore a head-mounted microphone to record audio of each of their productions. Additionally, we used a three-dimensional (3D) optical motion capture system (Motion Analysis 2012) to record the movement of the articulators during the motor learning task. Small, reflective markers were placed on the forehead, tip of the nose, corners of the lips, on the vermillion border of the center of the upper and lower lips, at the jaw gnathion, and on the right and left corners of the jaw below the lip corners (see Fig. 2A). The lower lip sensor was of interest for this study as it would reflect the combined movement of the lower lip and the jaw, and we hypothesized it would provide the greatest movement during the production of the nonwords. We used the head markers to remove head movement from the lower lip movement signal.

##### Retention

To examine how participants retained their newly acquired ability to produce the nonwords, they were asked to participate in a retention test ~ 24 h after their motor learning session. Each participant engaged in a recorded phone call in which they were given a live model of each nonword and were asked to repeat each ten times as quickly as they could. The nonwords were presented in the same order as they were given during the speech motor learning session. The recorded productions were later analyzed for production duration as described below. Due to poor signal quality, only durations were analyzed, as temporal measures of speech are more robust to noise in the acoustic signal than, for example, spectral or accuracy measures. This retention task was included to ensure that learning had occurred and was retained after a 24-h period (Schmidt et al. 2019).

#### Transcranial magnetic stimulation

**Fig. 3:**
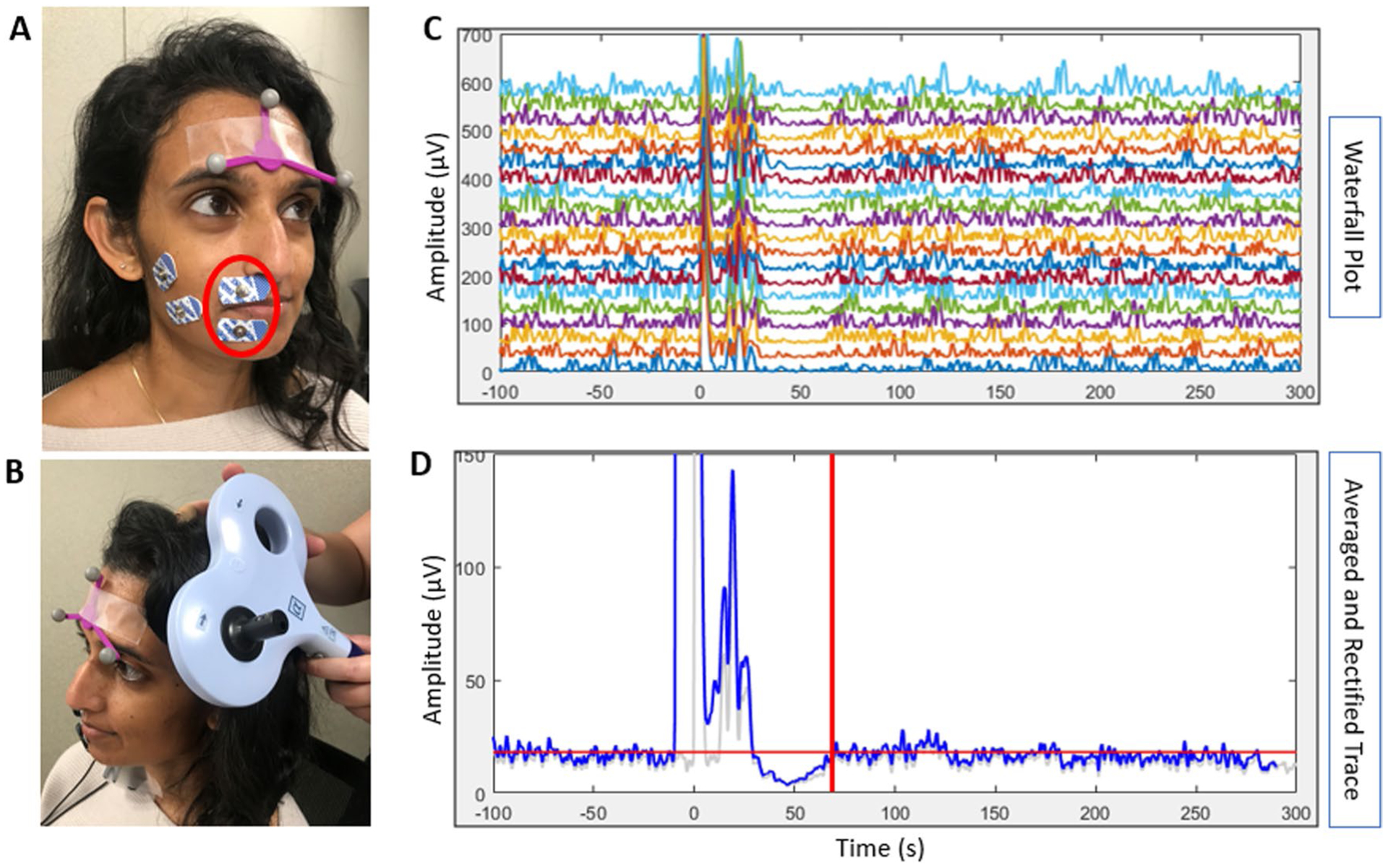
Transcranial magnetic stimulation cortical excitability data collection set-up and analysis. **A** Placement of the electromyography (EMG) electrodes and subject tracker. The lip electrodes used for analysis in this study are circled in red. Data collected from the electrodes on the masseter muscles were not used for the current study. **B** Orientation of the TMS coil for stimulation of the orbicularis oris muscle. **C** Waterfall plot showing 20 stimulations of the orbicularis oris region of motor cortex. **D** The result of averaging and rectifying the 20 traces in **C** is presented in blue and the one standard deviation trace is presented in light gray. The end of the cortical silent period (cSP) is marked with a red line

Procedures were followed similar to established methods of TMS measurement in the bulbar musculature (Adank et al. 2018; Möttönen et al. 2014; Samargia et al. 2014). Participants were seated in a comfortable, semi-reclined chair with arms and legs supported to facilitate relaxation, in a quiet environment. Pairs of disposable surface electromyography (EMG) electrodes were placed after vigorous cleansing of the skin with an alcohol prep pad. Electrodes were placed on the right first dorsal interosseous (FDI) muscle and the right upper and lower lip via manual palpation of the muscle belly (see Fig. 3A), and a ground electrode was placed behind the ear over the mastoid prominence. TMS was delivered through a 70-mm figure-of-eight coil connected to the Magstim 200^2^ stimulator (The Magstim Company Ltd, UK). All data were collected and stored on an iMac computer using a TMS neuronavigation system (Brainsight, Rogue Research Inc., Canada) to monitor real-time EMG activity and to provide the participants with visual EMG feedback for muscle contraction. The TMS coil was placed tangentially to the scalp with the handle in a posterior–anterior direction 45° to the mid-sagittal line of the head (see Fig. 3B; see similar TMS procedures in Chen et al. 2015, 2017). The subject tracker was attached to participants’ foreheads and a coil tracker was attached to the TMS coil to track stimulation site using the frameless stereotactic neuronavigation system (Brainsight, Rogue Research Inc., Canada).

##### Hotspot and active motor threshold determination

Using a template magnetic resonance imaging (MRI) brain scan in the neuronavigation system to guide the localization of the primary motor cortex (M1), the scalp site over the hand region of left M1 was approximated. Participants were instructed to relax their hand muscles and single-pulse TMS stimulation at 50% maximum stimulator output (MSO) was used to determine an individual FDI hotspot for each participant via visualization of a motor evoked potential (MEP) in the EMG signal. Once an MEP was observed, the experimenter moved the coil caudally on the left M1 to find the hotpot for the orbicularis oris muscle. Due to the difficulty of eliciting an MEP in facial muscles without active contraction (Iida et al. 2014; Lu et al. 2013; Watson et al. 2000), participants were instructed to contract their lip muscles by pressing their lips together at approximately 10% maximum voluntary contraction (MVC) during TMS stimulation. MVC was determined by instructing participants to press their lips together “as hard as they could”; the highest amount of EMG activity elicited was considered the MVC. Target contraction level via real-time EMG response visualization was thoroughly explained and participants practiced using it as feedback prior to data collection to ensure sustained contraction of the lips during stimulation. For determination of the lip hotspot, we began TMS stimulation at 60% MSO. Once an MEP was visualized, the coil was systematically moved in an approximate 1-cm grid to find the spot that elicited the highest MEP value with the lowest amount of stimulation, which was considered the hotspot for the orbicularis oris muscle. The established hotspot was used for all TMS testing.

The active motor threshold (AMT) was determined at the hotspot location and used to individualize the stimulus intensity for the cSP measurements. The AMT was defined as the lowest TMS intensity that elicited an MEP with a peak-to-peak amplitude of at least 100 microvolts (μV) in five out of ten consecutive trials with participants maintaining a slight contraction of ~ 10% MVC of the lip muscles during stimulation. The AMT was determined at baseline for each condition.

##### Cortical silent period

Cortical excitability of motor cortex associated with the orbicularis oris muscle was evaluated using the cSP. Specifically, the cSP reflects intracortical inhibition mediated by GABA_B_ receptors (Groppa et al. 2012). Stimulation was delivered at an intensity of 120% of the AMT for the majority of participants. AMT was re-established during the baseline phase for the second condition to account for potential variability over time and the % of MSO was adjusted if necessary. For three participants, 120% of the AMT did not elicit a visible cSP. For these participants, the lowest TMS intensity that elicited a consistent cSP was used for stimulation. Similarly, for two other participants, 120% of the AMT elicited a visible cSP, but a slightly higher intensity elicited a cleaner cSP, so this higher intensity was used throughout TMS testing. Single-pulse cortical stimulations were performed under sustained contraction (via pressing their lips together) of the lips at ~ 10% of participants’ maximum contraction, which was marked on the computer screen showing EMG feedback to allow participants to maintain a similar contraction level during each stimulation trial. Participants were instructed that consistency of contraction strength was the goal and that it must be maintained until they were instructed to relax. The single TMS pulse was applied approximately 1 s after initiation of lip contraction and participants were instructed to relax approximately 2 s after the pulse was delivered. Trials were disregarded during data collection when there was no baseline EMG activity, the participant did not maintain a targeted contraction level as instructed, or a cSP was not visualized.

##### Procedure

A flowchart of the experimental procedures for the TMS session is presented in Fig. 1B. Twenty trials of the cSP were collected pre and post each condition. The two conditions were the same as in the procedure for the speech motor learning task and there was a 20-min washout period between conditions to minimize risk of carry-over effects. The chewing and speech conditions were completed in the same order as for the speech motor learning task for each participant.

### Data analysis and outcome measures

A summary of all outcome measures considered in this study is presented in Table 1.

#### Speech motor learning task

##### Perceptual production accuracy

A trained research assistant was provided with the target of each nonword and listened to each of the 75 repetitions per condition per participant. The research assistant marked each of the eight syllables as correctly or incorrectly produced to derive *percent syllables correct* (%) for each repetition. When a participant repeated syllables in an incorrect order, these syllables were marked as incorrect. If a participant self-corrected, these syllables were marked as correct. Intra-rater reliability was measured by the same research assistant judging accuracy for 20% of the data, or four randomly selected participants, a second time on a second date to be compared to the initial accuracy judgements. Inter-rater reliability was measured by a second judge completing accuracy judgments for 20% of the data, or four randomly selected participants, which were later compared to accuracy calculations from the initial judge.

##### Acoustically derived production duration

Production duration was analyzed using Praat (Boersma and Weenink 2018). A text grid was used to mark the beginning and end of each repetition of the nonwords using the waveform and spectrogram. The beginning of each production was marked as the burst of the stop consonant and the end of each production was marked as the end of the acoustic energy for the nasal consonant (see Fig. 2B). After each repetition was marked in the text grid, a custom script was used to extract the *duration* [in seconds (s)] of each marked segment. Intra-rater reliability was conducted by the same research assistant completing the duration measurements for 20% of the data, or four randomly selected participants, a second time on a second date. Inter-rater reliability was conducted by a second judge completing the duration measurements for 20% of the data, or four randomly selected participants. Additionally, the ten productions recorded during the retention task for each participant in each condition were analyzed in the same way to derive production duration after a retention period of 24 h.

##### Kinematically derived lip speed and distance

The kinematic signals were analyzed using a software program called Cortex (Motion Analysis 2012). The distance from the right top head marker to the center of the lower lip (see Fig. 2C) was used to parse the kinematic signal for each repetition from trough (closing of the mouth for stop consonant) to trough (closing of the mouth for the nasal consonant). Using a custom script, the kinematic signal was cut into 75 individual repetitions, which were then run through a custom MATLAB program called Speech Movement Analysis for Speech and Hearing Research (SMASH; Green et al. 2013) to derive *average speed* [measured in millimeters/second (mm/s)] and *total distance* [measured in millimeters (mm)] of the lower lip across each repetition. Intra-rater reliability was measured by the same experimenter re-parsing the kinematic signal for 20% of the data, or four randomly selected participants, a second time on a second date. Inter-rater reliability was measured by a second judge parsing the kinematic signal for 20% of the data, or four randomly selected participants.

#### Transcranial magnetic stimulation

##### Cortical silent period

EMG signals were amplified and digitized according to established methods (Chen et al. 2017) and MATLAB codes were adapted from two previous studies (Fisher et al. 2016; Kuo et al. 2017). cSP was quantified offline and defined as the time from the TMS onset to the point when the interruption of EMG activity in the contracted muscle [measured in milliseconds (ms)] returned to baseline contraction levels (Kimberley et al. 2009). A decrease in cSP length would indicate decreased intracortical inhibition. EMG signals across the 20 stimulation trials were viewed in a waterfall plot (see Fig. 3C) and as an averaged and rectified trace across the 20 trials (see Fig. 3D). Then, a 10-ms moving window was applied to calculate the standard deviation (SD) of the EMG data at every ten ms to generate an SD curve of the signal (Chen et al. 2017). The average value of the SD curve 100 ms before the stimulus was calculated as the baseline contraction level. This value was then used to define the offset of the cSP when the signal returned to the baseline contraction level (see Fig. 3D). The point at which the averaged EMG activity returned to the baseline contraction level after the silent period was recorded as the offset. Consensus was reached between two investigators when uncertainty arose about cSP offset. When consensus could not be reached, a third investigator helped make the determination. Data for four of the 20 participants were removed due to unclear cSP endpoints, lack of silent period, and/or difficulty obtaining adequate EMG signal from the lip electrodes. Participants were removed by the consensus of the three investigators. Therefore 16 participants were included in the cSP analyses.

### Statistical analysis

All statistical analyses were completed in R (R Development Core Team 2013).

#### Measurement reliability

Measurement reliability for the outcomes of production accuracy, production duration, lip movement speed, and lip movement distance was completed with the psych package in R (Revelle 2019). Intra-rater reliabilities between the first and second measurements as well as inter-rater reliabilities for measurements completed by separate raters were assessed with two-way random single measures intraclass correlation coefficients (ICCs) used on consistency/absolute agreement (ICC 3,1).

#### Speech motor learning across conditions

Linear mixed effects models (LME) were used to examine the difference between conditions for each outcome variable (i.e., accuracy, duration, speed, and distance) across the 75 repetitions with the lme4 package in R (Bates et al. 2015). Repetition was used as a fixed factor and participant as a random factor to account for inter-individual variability in the intercept and slope of each participant’s performance. The difference of the rate of performance (on each outcome measure) over repetition for each condition was explored, as well as the mean performance (on each outcome measure) for each condition. The control condition was mapped to the intercept of each model to maximize interpretability and compare the other two conditions to it. Post hoc tests were conducted as appropriate to explore differences between the chewing and speech conditions. An alpha level of *p* < 0.05 was used for significance testing. For the retention task, we averaged the durations of the ten productions for each non-word and explored differences between the conditions with an LME containing participant as a random factor. We also performed Cohen’s *d* effect sizes with the effsize package in R (Torchiano 2020), to examine the magnitude of the duration difference between conditions after the retention period.

#### Cortical excitability

Paired *t* tests were used to evaluate statistical differences in cSP from pre- to post-continuous chewing and from pre- to post-continuous speech, as well as between the two conditions before each condition (pre-chewing and pre-speech) and after each condition (post-chewing and post-speech).

## Results

### Measurement reliability

Perceptual accuracy (measured in percent syllables correct) had good intra-rater reliability with a ICC of 0.87 (95% CI 0.86–0.88, *p* < 0.001) and excellent inter-rater reliability with an ICC of 0.94 (95% CI 0.94–0.95, *p* < 0.001). Reliability of production duration was found to be excellent with an intra-rater ICC of 0.99 (95% CI 0.99–1.00, *p* < 0.001) and an inter-rater ICC of 1.00 (95% CI 0.99–1.00, *p* < 0.001). Reliability of the kinematic measures was also adequate: average speed of the lower lip had an intra-rater ICC of 0.92 (95% CI 0.91–0.92, *p* < 0.001) and an inter-rater ICC of 0.81 (95% CI 0.80–0.83, *p* < 0.001); total distance of the lower lip had an intra-rater ICC of 0.97 (95% CI 0.97–0.98, *p* < 0.001) and an inter-rater ICC of 0.93 (95% CI 0.92–0.94, *p* < 0.001).

### Speech motor learning task across conditions

#### Production accuracy

**Fig. 4:**
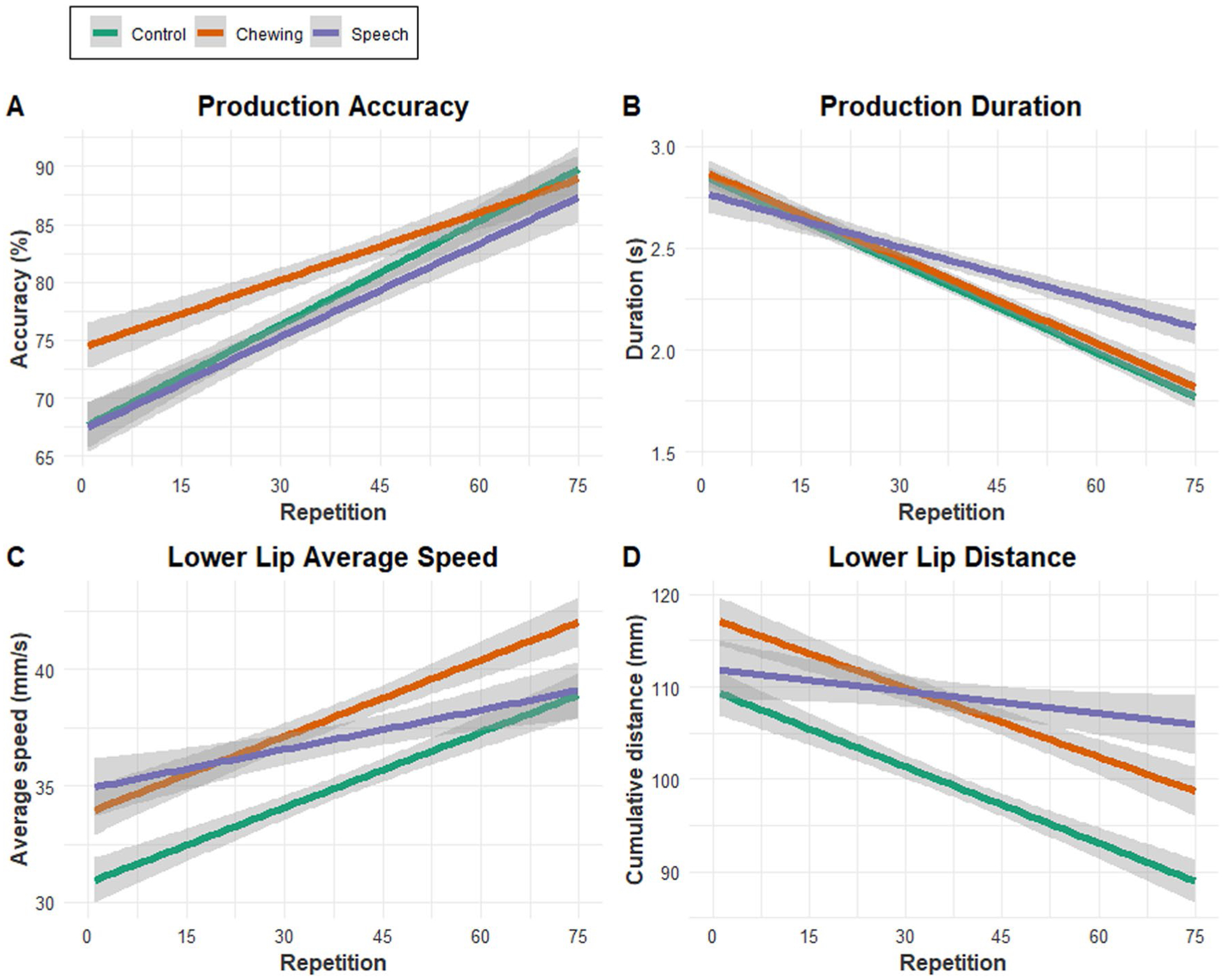
Linear models of **A** Production accuracy; **B** production duration; **C** average speed of the lower lip; and **D** total distance of the lower lip across repetitions of the speech motor learning task in the three conditions. The gray-shaded areas represent the 95% confidence interval around the linear models

There was a significant overall linear trend of production accuracy over repetition (*t* = 14.18, *p* < 0.001); fixed-effect estimates for the condition by repetition interaction revealed differential rates of change in accuracy across conditions. Specifically, the control and speech conditions had the same intercept (i.e., baseline score; *p* = 0.91), but the chewing condition had a significantly higher baseline score than the other two conditions (*p* < 0.001). The control and speech conditions had the same rate of accuracy change across repetitions (*p* = 0.31) and the chewing condition had a significantly less steep slope across repetitions than the other two conditions (*p* < 0.001). Figure 4A displays production accuracy averaged across participants for each repetition across the three conditions as linear models to allow for better visualization of the data.

#### Production duration

There was a significant overall linear trend of production duration over repetition (*t* = − 21.50, *p* < 0.001); fixed-effect estimates for the condition by repetition interaction revealed differential rates of change in duration across conditions. Specifically, the control and chewing conditions had the same baseline duration (*p* = 0.59), but the speech condition had a slightly shorter baseline duration than the other two conditions (*p* = 0.04). The control and chewing conditions had the same rate of production change across repetitions (*p* = 0.71) and the speech condition had a significantly less steep slope across repetitions than the other two conditions (*p* < 0.001). Figure 4B displays production duration averaged across participants for each repetition across the three conditions.

For the duration of the productions after the retention period, there was a main effect of condition (*p* < 0.001) and post hoc tests revealed significant differences between all conditions (i.e., control and chewing *p* = 0.007; control and speech *p* < 0.001; and chewing and speech *p* = 0.021). However, Cohen’s *d* effect sizes were small or negligible for all of these contrasts (i.e., 0.42, 0.28, and − 0.19, respectively), with the control condition having the shortest durations (mean = 2.16 ± 0.62), followed by the chewing condition (mean = 2.34 ± 0.66 s). The speech condition had the longest durations (mean = 2.49 ± 0.93 s).

#### Lower lip average speed

There was a significant overall linear trend of lower lip average speed over repetition (*t* = 12.91, *p* < 0.001); fixed-effect estimates for the condition by repetition interaction revealed differential rates of change in average speed across conditions. Specifically, the chewing and speech conditions both had faster baseline speeds than the control condition (both *p* < 0.001). The control and chewing conditions had the same rate of average speed change across repetitions (*p* = 0.65) and the speech condition had a significantly less steep slope across repetitions than the other two conditions (*p* < 0.001). Figure 4C displays speed of the lower lip (mm/s) averaged across participants for each repetition across the three conditions.

#### Lower lip distance

There was a significant overall linear trend of lower lip distance over repetition (*t* = − 10.84, *p* < 0.001); fixed-effect estimates for the condition by repetition interaction revealed differential rates of change in distance across conditions. Specifically, the chewing and speech conditions both had larger baseline distances than the control condition (*p* < 0.001 and *p* = 0.02, respectively). The control and chewing conditions had the same rate of average speed change across repetitions (*p* = 0.76) and the speech condition had a significantly less steep slope across repetitions than the other two conditions (*p* < 0.001). Figure 4D displays distance of the lower lip (mm) averaged across participants for each repetition across the three conditions.

### Cortical excitability

There was no significant change in cSP from pre-chewing (mean = 94.79 ± 36.66 ms) to post-chewing (mean = 95.63 ± 40.15 ms); *t*(15) = − 0.36, *p* = − 0.73; or from pre-speech (mean = 99.55 ± 41.22 ms) to post-speech (mean = 99.5 ± 40.85 ms); *t*(15) = 0.02, *p* = 0.99. Paired *t*-tests also demonstrated that cSP length was not statistically different between the pre-chewing (mean = 94.79 ± 36.66 ms) and pre-speech (mean = 99.55 ± 41.22 ms) measurements; *t*(15) = − 1.26, *p* = 0.23; or between the post-chewing (mean = 95.63 ± 40.15 ms) and post-speech measurements (mean = 99.5 ± 40.85 ms); *t*(15) = − 1.00, *p* = 0.33.

## Discussion

The current study investigated the across-trial changes in speech performance during a challenging eight-syllable non-word repetition task and the effects of continuous oromotor activity (i.e., 10 min of chewing and speech) on task performance and retention. Our hypothesis was that neuroplastic changes induced by continuous chewing and speech prior to the nonword repetition task would impact subsequent speech performance and be associated with reduced inhibition, as measured by TMS. The context for this study is the emerging literature suggesting that sustained motor activities induce neuroplastic changes that support learning and motor rehabilitation (Fisher et al. 2008; Hirsch et al. 2016; Petzinger et al. 2013). The primary findings of this work were the following: (1) speech accuracy and performance improved significantly across repetitions of the novel speech motor learning task; (2) speech accuracy and performance during the nonword repetition task was enhanced when it followed 10 min of continuous chewing; (3) in contrast, speech accuracy and performance was degraded when it followed 10 min of continuous talking; and (4) condition effects on task performance were not associated with detectible changes in cortical excitability as measured by the cSP of lip motor cortex.

The across-trial changes in speech accuracy and performance during the control condition provided a reference for evaluating the potential facilitating or interfering effects of continuous chewing and speaking on the speech motor learning task. During the control condition, production accuracy steadily increased over the repetitions while production duration decreased, findings that agree with previous work on speech motor learning (Richtsmeier and Goffman 2015; Sadagopan and Smith 2013; Sasisekaran et al. 2010; Whitfield and Goberman 2017a). Articulation accuracy was arguably the primary goal of the task as participants were verbally instructed to attend to accuracy before attending to speed. Because precision is the goal of many speech motor learning tasks, measures of articulatory accuracy have been used as primary outcome measures in previous studies of speech motor learning (Buchwald et al. 2019; Kaipa 2016). Improvements in accuracy coincided with refinements in lip movement across repetitions, including a decrease in extent and increase in speed. This reduction of lip movement is consistent with previous research demonstrating the economization of articulator movements adopted by neurologically healthy speakers. When instructed to increase speech rate, for example, healthy individuals tend to reduce the extent of articulator displacement rather than increase movement speed (Eshghi et al. 2019; Mefferd and Green 2010; Simione et al. 2018; Westbury and Dembowski 1993). Additionally, children increase their speech rate throughout development by minimizing lip and jaw displacements rather than increasing their movement speed to achieve a faster speech rate (Nip and Green 2013); when learning novel stimuli, healthy participants minimize the extent of movement as much as possible without sacrificing achievement of task goals, i.e., accuracy/precision (Lindblom 1990). Overall, findings from the control condition (1) demonstrate that across-trial improvements in speech performance on this novel speech task are consistent with findings from previous work, and (2) provide an empirical basis for evaluating the potential effects of continuous chewing and speech activity on speech motor learning.

The retention task provides evidence that the novel learned nonwords were retained after 24 h, as durations were shorter at the retention period, on average, than they were before any learning had occurred (i.e., production durations at early repetitions in Fig. 4B). Speech performance after the retention period, however, was not condition-dependent. Thus, the positive effect of chewing on speech performance in this study was observed only during practice. The condition effects observed during practice did not appear to transfer to long-term learning, which may have been due to the “low dose” of the chewing activity during the experiment, or to the short-interval, one-session of practice. We view this finding as tentative because the remote administration of the retention task via telephone only allowed us to measure production duration, excluding the measurement of speech accuracy and lip kinematics.

### Continuous chewing activity enhanced performance on the speech motor learning task

Compared to the control condition, there was a significant enhancement of speech performance following 10 min of gum-chewing. The priming effect of chewing on speech production is supported by the observation that accuracy differences between the conditions were apparent during initial trials of the learning task (see Fig. 4A), and that faster lip movement speeds and larger distances were maintained throughout the learning task (see Fig. 4C, D, respectively). Although the accuracy benefits in the chewing condition are not sustained throughout the task, it is unclear if this should be attributed a short modulatory window produced by the chewing activity, or ceiling effects occurring in the other conditions. The other outcome measures provide information regarding how the primary outcome of accuracy was achieved. Production duration did not differ between the chewing and control conditions throughout the task; however, lip excursions were larger (see Fig. 4D) and average speeds were faster (see Fig. 4C) during the chewing condition than during the control condition. Thus, although chewing induced more accurate productions, participants did not use the most efficient movement strategy. The latter would have involved minimizing articulatory excursions rather than increasing them. This pattern of articulation—increased accuracy and excursions—has been described as hyperarticulation, a ‘high-energy’ mode of talking that is intended to maximize accuracy and clarity (Lindblom 1990). Overall, the improved accuracy achieved early in the chewing condition, along with faster lip movements that were maintained across the duration of the task, suggest improved performance in this condition.

#### Hypotheses for improved speech performance following a period of chewing

As discussed earlier, a robust literature exists on the beneficial effects of chewing on the performance of cognitive tasks (Allen and Smith 2011; Onyper et al. 2011; Tucha and Koerts 2012). The enhanced performance we observed for the chewing condition is consistent with studies suggesting links between motor activity, cortical hyperexcitability, and enhanced learning (Iida et al. 2014; Kothari et al. 2011; Svensson et al. 2003). For example, Baad-Hansen et al. (2009) found positive associations between changes in corticomotor excitability of tongue M1 and success rate in performing a tongue-training task involving protrusion of the tongue against a force plate. However, we did not find evidence of change in the cSP in our TMS experiment. Specifically, we measured intra-cortical inhibition of M1, rather than MEP excitability. We elected to use the cSP as the measure of interest because, compared other TMS measures such as MEPs or paired pulse measures, it is less affected by muscle activation levels and is highly reliable (Chen et al. 2018). There are several possible explanations for the lack of cSP change, despite behavioral changes: (1) chewing may not directly modulate the excitability or inhibition of M1, or in particular, the lip region of M1 (see Kubo et al. 2013); (2) the dosage of chewing used in our experiment (10 min) may not have been sufficient to induce changes M1 (Allen and Smith 2011; Fisher et al. 2016; Svensson et al. 2006); and (3) the wide variability of baseline cortical excitability seen in our young, healthy participants may not allow for the ability to see within-condition differences, at least at a group level. Future work could examine individual trends in cortical excitability following various oromotor behaviors. In addition, an increased dose of activity may be necessary to produce larger and longer-lasting performance effects. Increased dosage may take the form of increased time engaged in the motor behavior (Allen and Smith 2011), a greater number of trials (i.e., pacing the chewing task), or performing the behavior against resistance (i.e., chewing a harder substance) (Tramonti Fantozzi et al. 2017).

The disparity between the current findings and previous findings of strong associations between cortical excitability and performance of non-speech tasks may underscore the uniqueness of speech as a motor act (Kent 2004). It is well-known that successful speech production relies on a multitude of brain regions aside from primary motor cortex, including pre-motor cortex, supramarginal gyrus, basal ganglia, frontal operculum, somatosensory cortex, and posterior superior temporal cortex (Segawa et al. 2015; Shum et al. 2011). Successful completion of the novel speech learning task in this study relies on extramotor processes such as working memory and attention (Ito et al. 2020; van Zelst and Earle 2020). Therefore, a secondary, but related hypothesis for the improved performance on the speech learning task in the chewing condition is that chewing creates a hyper-vigilant state that would be measurable in extramotor cortical regions. For example, increased excitability in frontal regions could improve facilities such as vigilance, alertness, or focus that could help to explain the improved speech performance. In fact, several authors have found positive effects of chewing on memory and recall (Allen and Smith 2011; Hirano et al. 2008; Stephens and Tunney 2004), which may explain the improved performance seen in the chewing condition of the current study. Improved performance on the speech motor learning task following a period of continuous chewing may also provide evidence for transference, or the premise that plasticity-inducing behaviors must be similar to the behavioral change of interest (Ludlow et al. 2008). Although transference between different oral motor behaviors, such as speech and swallowing (McFarland and Tremblay 2006), has been debated in the speech-language literature, information gained from the current research provides new insights into across-system interactions.

#### The priming effect of chewing behavior on speech performance does not support the use of non-speech oromotor exercises in speech therapy

On the surface, the suggestion that chewing enhances speech performance appears to parallel the widely debated and controversial suggestion that non-speech oromotor exercises (NSOMEs) can enhance speech therapy outcomes (Lof and Watson 2008; Maas 2017; Mccauley et al. 2009; Ruscello and Vallino 2020). Our hypothesized pathway of causation between chewing and speech performance, however, does not involve a direct transfer of one motor skill to the other, but rather a time-dependent enhancement of supporting neural processes such as attention, memory, and executive function. Thus, rather than chewing serving to improve speech production by training the oral muscles to move in a particular way, which is the premise of many researchers and clinicians who have used NSOMEs (Forrest 2002; Lof and Watson 2008; Strode and Chamberlain 1997), we propose that the current findings more likely demonstrate the ability of chewing (or another specific motor task) to prime the brain via extramotor processes that, under the right conditions, could enhance the ability to learn.

### Continuous speech activity interfered with performance on the speech motor learning task

A second finding of the current investigation was the absence of performance improvements across repetitions that followed the continuous speech condition. The observed changes during the control and chewing condition were consistent with what would be expected if speakers were iteratively tuning the intrinsic dynamics of the articulatory control system across repetitions. Although initially, production accuracy and duration during the speech condition were indistinguishable from those in the control condition, the speech condition had a significantly shallower rate of improvement. By the last repetition, production durations were significantly longer in the speech condition than during the control or chewing conditions. Similarly, the distance traveled by the lower lip did not change in the speech condition, suggesting that movement economization was not implemented across-trials. The kinematic results suggest dysregulation of movement under the speech condition. Although at the beginning of the speech motor learning task, average lip speed was increased in the speech condition relative to the control condition, this increase was not adaptive—it did not lead to improved accuracy or shortened durations and thus, did not help participants achieve the goal of the task. Motor learning interference and dysregulation has been observed under other neuromodulatory techniques (Simione et al. 2018; Wach et al. 2013). The authors of these studies have considered at least three possible explanations:

1. Neuromodulators are known to either facilitate or impede learning/behavior. Research on external neuromodulation techniques such as tDCS, for example, has shown changes in behavior that have been thought to be due to upregulation or downregulation (i.e., excitation or inhibition; enhancement or suppression), respectively, of neural circuitry (The International Neuromodulation Society 2016). The excitatory effects of anodal stimulation are primarily thought to strengthen or improve behavior, while the inhibitory effects of cathodal stimulation have traditionally been considered for the hindrance of behavior (Nitsche and Paulus 2000); however, these traditional uses of tDCS have been challenged by more recent work suggesting that excitation of certain brain areas can cause dysregulation and maladaptive movement patterns (Jacobson et al. 2012; Pirulli et al. 2014; Simione et al. 2018). A few authors have reported on the impeding effects that engaging in a simultaneous behavior can have on neuromodulation (Antal et al. 2007; Horvath et al. 2014; Quartarone et al. 2005), though the potential of specific motor behaviors alone to impede learning requires more investigation.
2. Cognitive resources needed to perform the speech motor learning task may have been diminished by the period of continuous speech, consistent with the theory of resource allocation (Kahneman 1973). The finding of impeded performance was more pronounced at the end of the task (i.e., in repetitions 40–75), where performance in the speech condition diverged from the control and chewing conditions. The divergence of performance in the speech condition late in the task may indicate the role of diminished resources/fatigue on speech performance following continuous speech production (Herath et al. 2001).
3. A final hypothesis for the impaired speech performance following speech is repetition suppression, which has been defined as “repeated experience with the same… stimulus leads to both short and long-term suppression of neuronal responses…” (Desimone 1996, p. 13494). Thus, repeated use of the same neural circuitry can lead to degraded, or lessened neural responses needed for a specific task (Kallioniemi et al. 2015). Therefore, engaging in a 10-min task involving speech production may have suppressed the neural responses that were required for performance on the novel speech learning task, resulting in degradation of speech performance.

## Strengths, limitations, and future directions

The number of experimental paradigms for testing speech motor learning is limited (see Adams and Page 2000; Buchwald et al. 2019; Kaipa et al. 2017; Sadagopan and Smith 2013; Steinberg Lowe and Buchwald 2017; Whitfield and Goberman 2017a, b for examples of such paradigms). However, speech learning tasks are greatly needed to identify optimal speech therapy parameters (e.g., feedback and practice conditions) and to explore the efficacy of adjuncts designed to facilitate speech outcomes including neuromodulation techniques such as tDCS and rTMS. Young, neurologically healthy individuals have historically performed well on novel speech tasks which belies the examination of speech learning processes. Overall, the novel speech motor learning task developed for this study was effective for inducing substantive performance improvement trajectories. Several strengths of this task include a lack of ceiling effects early in the task, the ability to apply a variety of outcome measures, and relative ease of instruction/implementation. Several weaknesses of task-implementation include some ceiling effects at the end of the task (i.e., as production accuracy levels were matched for all three conditions by the end of the 75 repetitions), the remote data collection after the retention period which did not allow for the computation of all outcome measures (i.e., perceptual accuracy and kinematics), and potential influences of working memory unrelated to pure motor control. Future work comprehensively investigating speech performance after a retention period is warranted to enhance our understanding of true speech learning on this task.

It is possible that the greater accuracy at the beginning of the chewing condition as compared to the control condition could have been the result of order effects. We selected the control condition to always occur first to avoid carry-over effects of the oromotor behaviors on the control condition. The reduced accuracy in the control condition relative to the chewing condition, particularly during early repetitions, may be due to the novelty of the task rather than the hypothesized priming effects of chewing. Order-effect concerns are mitigated by the finding of higher lip speeds that persist across the duration of the task in the chewing condition.

Lack of change in the cSP following chewing and speech may be the result of small participant numbers, inherent neurophysiological variability in the participants, low dosage of the modulating activity (i.e., only 10 min of chewing and speech), or measurement of an off-target neurophysiologic marker or in an off-target muscle. Future work could explore these possibilities by examining individual trends in TMS outcome measures, increasing sample sizes, attempting new methods for obtaining various TMS measurements of the oromotor neuropathways, examining cortical excitability of other orofacial muscles such as tongue or masseter, or increasing the dose of oromotor activity (i.e., by increasing the amount of time or making the tasks more challenging). Nevertheless, the current study demonstrates the feasibility of completing cSP measurements of lip M1 with TMS in young, neurologically healthy individuals.

## Conclusions

This exploratory study provides evidence for oromotor activity-induced changes in speech motor performance in healthy individuals. Specifically, a period of continuous chewing had a beneficial effect on subsequent speech performance, whereas a period of continuous speech impeded subsequent speech performance. The observed behavioral and biomechanical changes in speech motor performance following continuous oromotor activities provide a strong rationale for continued exploration of the rehabilitation implications in a variety of speech impaired populations. The finding of impeded performance following sustained speech may have implications for the structuring of speech therapy sessions, including the importance of task selection and timing of activities. For example, placing demanding tasks at the beginning of a therapy session may be detrimental for performance on subsequent tasks, especially if they involve similar neural circuitry or cognitive resources.

## Figures and Tables

**Table 1:** Outcome measures used in the current study

Task	Type of analysis	Measure	Unit
Speech motor learning	Perceptual	Production accuracy	Percent syllables correct (%)
	Acoustic	Production duration	Milliseconds (ms)
	Kinematic	Lip movement speed (average)	Speed [millimeters/second (mm/s)]
		Lip movement distance (total)	Distance [millimeters (mm)]
Transcranial magnetic stimulation	Neurophysiologic	Cortical silent period (cSP)	Milliseconds (ms)

## Data Availability

The data that support the findings of this study are available from the corresponding author JRG upon reasonable request.
